# Novel ADC and γδ T cell engager targeting CDH17 for the therapy of gastrointestinal cancers

**DOI:** 10.1093/abt/tbag012

**Published:** 2026-03-12

**Authors:** Mingcan Yu, Guangmao Mu, Peng Chen, Honglei Bi, Fulai Zhou, Zhengxia Zha, Sheng Huang, Hao Jiang, Ying Jin, Yuanting Chen, Mark L Chiu, Di Zhang

**Affiliations:** Tavotek Biotherapeutics Inc., Ambler, PA 19002, United States; Tavotek Biotherapeutics Inc., Suzhou, 215000, China; Tavotek Biotherapeutics Inc., Suzhou, 215000, China; Tavotek Biotherapeutics Inc., Suzhou, 215000, China; Tavotek Biotherapeutics Inc., Suzhou, 215000, China; Tavotek Biotherapeutics Inc., Suzhou, 215000, China; Tavotek Biotherapeutics Inc., Suzhou, 215000, China; Tavotek Biotherapeutics Inc., Suzhou, 215000, China; Tavotek Biotherapeutics Inc., Suzhou, 215000, China; Tavotek Biotherapeutics Inc., Ambler, PA 19002, United States; Tavotek Biotherapeutics Inc., Ambler, PA 19002, United States; Tavotek Biotherapeutics Inc., Ambler, PA 19002, United States

**Keywords:** CDH17, ADC, γδ T cell, T cell engager, gastrointestinal cancer

## Abstract

**Background:**

Cadherin-17 (CDH17) is a cell-adhesion molecule physiologically expressed along the intestinal epithelial tight junctions. Aberrant overexpression of CDH17 in gastrointestinal (GI) cancers promotes tumor growth and metastasis and is associated with poor patient prognosis. Due to its restricted expression in normal tissues and strong association with malignancy, CDH17 represents an emerging therapeutic target for GI tract cancers.

**Methods:**

A high-affinity anti-CDH17 monoclonal antibody (TAVO307) was generated and conjugated with auristatin-derived cytotoxic payloads to obtain CDH17-directed antibody–drug conjugates (ADCs). In parallel, a VHH antibody capable of activating γδ T cell receptors from both Vδ1 and Vδ2 subsets was identified and combined with the anti-CDH17 antibody to generate CDH17-targeted T cell engager (TCE) to recruit γδ T cells, which are abundant in the intestinal mucosa and play a critical role in tumor immunosurveillance. An attenuated interleukin-15 (IL-15) fused with the IL-15 receptor α sushi domain was further incorporated on TCE to enhance γδ T cell expansion and activation.

**Results:**

CDH17-based ADCs exhibited potent and selective cytotoxicity in multiple GI cancer cell lines and significant tumor regression in xenograft models. The CDH17 γδ TCE induced tumor antigen-dependent γδ T cell degranulation and redirected both Vδ1 and Vδ2 T cells to effectively kill CDH17-expressing cancer cells. IL-15 fusion further augmented γδ T cell expansion and activation.

**Conclusions:**

Both CDH17-targeted ADCs and γδ TCEs demonstrated promising potency and efficacy to control GI cancers. They could offer complementary therapeutic options that could be used in combination therapy.

## Teaser

Statement of Significance We developed two novel complementary therapeutic modalities targeting an emerging gastrointestinal tumor-associated antigen, CDH17: an antibody–drug conjugate that exhibited potent tumor cytotoxicity and a γδ T cell engager that recruited and activated both Vδ1 and Vδ2 subsets of γδ T cells to effectively kill CDH17-expressing cancer cells.

## Introduction

The cadherin family comprises calcium-dependent adhesion molecules that preferentially interact with themselves to connect cells in order to maintain tissue architecture, polarity, and barrier integrity. Among them, cadherin-17 (CDH17), also known as liver-intestine cadherin or intestinal peptide-associated transporter (HPT-1), is an atypical cadherin family member with an extracellular domain with 7 cadherin domains, a single hydrophobic transmembrane domain, and a short C-terminal cytoplasmic tail [1]. CDH17 is expressed predominantly in the epithelial cells lining the intestinal tract, including the duodenum, jejunum, ileum, colon, and certain pancreatic excretory ducts, while it is not detected in normal tissues of the kidney, lung, liver, brain, adrenal gland, or skin [2, 3]. CDH17 is localized to the apical and lateral membrane domains of intestinal epithelial cells in proximity to tight junction structures. It mediates intercellular adhesion, contributing to epithelial barrier integrity [4].

In contrast, CDH17 is aberrantly overexpressed in multiple gastrointestinal (GI) tract malignancies, including colorectal, gastric, liver, esophageal, pancreatic, ovarian, uterine, and prostate cancers [2, 3, 5–8]. It has been identified as a biomarker to predict GI tract cancers with a high sensitivity [2, 9, 10]. Besides, CDH17 plays an important role during the process of tumor growth, invasion, and metastasis as an oncogene by multiple mechanisms. It promotes tumor growth in liver carcinoma by regulating the Wnt/β-catenin signaling pathway [11, 12]. It regulates the activity of the Ras/Raf/MEK/ERK pathway for cell proliferation in gastric cancer [13]. CDH17 also interacts with α2β1 integrin to regulate cell proliferation and adhesion in colorectal cancer cells, causing liver metastasis [14]. Moreover, high CDH17 expression correlates with shorter survival and disease progression in colorectal, gastric, and liver cancer patients [14–17].

In GI cancers, the development of therapeutic drugs has been constrained by the scarcity of truly tumor-restricted antigens. The low expression in healthy tissues compared to tumor overexpression makes CDH17 an attractive tumor-associated antigen for selective targeting. Besides, targeting CDH17 may have an even more favorable therapeutic index due to the relative inaccessibility of the tight junction structure, which shields T/NK cells and restricts macromolecules from attacking CDH17 in physiological tissues [18], while the normal epithelial barrier architecture is disrupted in GI tract malignancies, which facilitates tumor targeting. One of the early drugs targeting CDH17 was BI 905711, a novel tetravalent bispecific antibody targeting both CDH17 and TRAILR2 (the tumor necrosis factor-related apoptosis-inducing ligand receptor 2), exhibiting potent and selective antitumor activity [19]. In recent years, ADC has emerged as a powerful precision oncology tool by coupling antibody specificity with the cytotoxic potential of small-molecule payloads. Several CDH17-targeting ADC molecules showed potent tumor cytotoxicity in preclinical studies, with some of them, including TORL-3-600, 7MW4911, and AMT-676, in early clinical investigation [20]. Besides ADC, CAR-T cells targeting CDH17 exhibited potent suppression of neuroendocrine tumors and colorectal cancer liver metastases without toxicity to normal tissues [18, 21]. CDH17-targeting CAR-NK cells demonstrated potent cytotoxicity to GI cancers as well [22]. Bispecific T cell engagers (TCEs) targeting CDH17 and CD3, including cabotamig and LBL-054-CD3, were reported to have good efficacy in the elimination of pancreatic and colorectal cancer cells and were evaluated in clinical trials. Together, these developments suggest that CDH17 is transitioning from a largely underexplored antigen to one of practical therapeutic value.

However, common problems of engaging T cells, particularly the dominant αβ T cell population, in tumor elimination are the risk of having associated cytokine release syndrome (CRS) and immune effector cell-associated neurotoxicity syndrome (ICANS) [23]. These risks limit the realization of an efficacious dosing because of toxicity to the patients. One promising alternative approach to overcome these risks is to leverage γδ T cells, a nonconventional subset of T cells, in tumor elimination [24]. γδ T cells exhibit a different cytokine activation with lower autocrine amplification and less activation of monocytes/macrophages linked to CRS pathogenesis [25, 26]. Therefore, one of the major advantages of γδ T cell recruitment in tumor immunotherapy is to be less prone to cytokine storm often associated with the CD3-based TCE activation of αβ T cells. A clinical trial evaluating allogeneic Vγ9Vδ2 T cell immunotherapy of late-stage lung or liver cancer reported fewer and lower-grade CRS events relative to those studies with CD3-based TCE [27].

In contrast to αβ T cells, the T cell receptor (TCR) of γδ T cells is composed of γ and δ TCR chains [28]. γδ T cells are innate-like immune cells that detect tumors and pathogen-infected cells through innate natural killer (NK) receptors without the obligate requirement for cognate tumor associated antigen presentation on major histocompatibility complex molecules. In humans, γδ T cells represent 1%–10% of total CD3^+^ T cells and express a combination of 7 different γ chains paired with 4 δ chains [29, 30]. The Vγ9Vδ2 T cell subset is the predominant one in peripheral blood by sensing small, phosphorylated metabolites (phosphoantigen [pAg] molecules) arising from stress conditions like infection or malignant transformation [31, 32]. The Vδ1 T cell subsets, with δ1 paired with different γ chains, recognize different ligands, including pathogenic and self-lipids presented by CD1d [33, 34], to combat malignant and infected cells by residing in tissues that include the skin, large intestine, lung, and liver [30, 35]. Besides, γδ T cells, dominantly the Vδ1 T cell subsets, are found in various tumors like rectal cancer, breast cancer, or pancreatic cancer [36–38]. By direct cytotoxicity, cytokine secretion (IFN-γ, TNF), and modulation of the tumor microenvironment, γδ T cells bridge the innate and adaptive immune responses and provide a first line of immunosurveillance against aberrant cell growth and infectious diseases. Indeed, γδ T cells were identified as the prognostically most favorable immune cell subset in tumor infiltrates from 18 000 tumors across 39 malignancies [39]. Higher γδ T cell frequency in tumors from cancer patients correlates with better clinical outcomes in different malignancies [40–43].

While accounting for fewer than 5% of peripheral blood T cells, γδ T cells are present in much higher numbers (10%–100%) in epithelial tissues of the GI tract, representing the major population of intraepithelial lymphocytes [44, 45]. These epithelial γδ T cells are key contributors to epithelial homeostasis, repair, and barrier surveillance of infection. Prognostic studies revealed that colorectal and gastric cancer patients containing abundant intratumoral γδ T cells had a significantly longer 5-year disease-free survival rate, suggesting their efficacy in controlling GI cancers [41, 42]. Clinical trials with adoptive transfer of γδ T cells into patients further demonstrated impressive efficacies in colorectal, gastric, and esophageal cancers [46, 47]. It is thereby speculated that recruiting γδ T cells that infiltrated GI tract tumors is a viable approach to treating these cancers.

In this study, we showed two complementary strategies to exploit CDH17 as a therapeutic target in GI cancers: ADCs directed to CDH17 conjugated with potent cytotoxic payloads and a γδ TCE that redirected γδ T cells to CDH17-expressing tumor cells. The mechanisms of action of these CDH17 molecules were demonstrated by binding, antitumor activity, γδ T cell activation, and *in vivo* tumor growth inhibition. In addition, we discussed mechanistic insights, safety considerations, and translational potential.

## Materials and methods

### Antibody expression and purification

Gene fragments encoding antibody heavy chain (HC) and light chain (LC) were synthesized by IDT (Coralville, IA) and cloned into pcDNA3.4 expression vector (ThermoFisher, A14697). Antibody HC and LC constructs (1:3 molar ratio) were co-transfected into Expi293F cells by the Expifectamine293 transfection kit (ThermoFisher, A14525). Cells were incubated for 5 days at 37°C and the supernatant was harvested and purified by affinity chromatography over a MabSelect SuRe column (Cytiva, 29049104) followed by a desalting column. Protein concentration was determined by Nanodrop (ThermoFisher) with UV absorbance at 280 nm. The null antibody used in assays referred to the antibody against human HIV virus envelope protein gp120.

### Conjugation monomethyl auristatin F or monomethyl auristatin E on CDH17 antibody

Monomethyl auristatin F (MMAF) was conjugated to the anti-CDH17 antibody TAVO307 by the oYo-Link antibody conjugation kit according to the manufacturer’s instructions (AlphaThera, oYo-Link VcMMAF). The efficiencies of conjugation by photo-crosslinking were verified by SDS-PAGE under reduced conditions. Monomethyl auristatin E (MMAE) was conjugated by cysteine-based coupling. Briefly, TAVO307 antibody was reduced by Tris(2-carboxyethyl)phosphine (ThermoFisher, 77720) at 30°C for 2 h and then reacted with MC-Val-Cit-PAB-MMAE (MedChemExpress, HY-15575) for another 2 h, followed by the removal of unreacted free drugs by PD MidiTrap G-25 desalting column (Cytiva, 28-9180-08). The conjugated antibody was analyzed by hydrophobic interaction chromatography (HIC) for drug-to-antibody ratio (DAR) and also by size exclusion chromatography (SEC).

### Bispecific antibody γδ TCE generation

Bispecific antibodies TAVO307 × VHH371 and TAVO307 × VHH371_IL15 as γδ TCE were generated by the knobs-into-holes technology [48]. S354C and T366W mutations were introduced into the IgG1 Fc of the TAVO307 antibody, and Y349C, T366S, L368A, and Y407V mutations were introduced into the IgG1 Fc of VHH371 with or without C-terminus fusion with the sushi domain and IL-15 with S7T and I68K mutations. Additionally, the IgG1 Fc of the bispecific γδ TCE contained the L234A and L235A mutations. Heavy chain and light chain constructs encoding TAVO307 antibody and VHH371 or VHH371_IL15 IgG1 chain were co-transfected into Expi293F cells by the Expifectamine293 transfection kit (ThermoFisher, A14525). Cells were incubated for 5 days at 37°C and the supernatant was harvested and purified by affinity chromatography over a MabSelect SuRe column followed by a desalting column. Protein concentration was determined by Nanodrop with UV absorbance at 280 nm.

### CDH17 binding assay

Recombinant human, cynomolgus monkey, and mouse CDH17 (AcroBiosystems, CA7-H52H3, CA7-C52H3, CA7-M52H5) were coated at the 1 μg/ml concentration in DPBS pH 7.2 on a Maxisorp 96-well plate. After blocking, serial dilutions of test antibody were applied to the plate and incubated for 2 h at room temperature by shaking. After washing the plates four times with washing buffer, HRP-conjugated anti-human IgG secondary antibody (Jackson ImmunoResearch, 109–036-098) was added for the detection. The plate was washed four times with wash buffer, then TMB (3,3′,5,5′-tetramethylbenzidine) substrate (ThermoFisher, 34029) was added to the plate for color development. Optical densities were determined with a SpectraMax i3X plate reader (Molecular Devices) at a 450 nm wavelength.

For cell binding, serial dilutions of antibody were applied to the CDH17-expressing tumor cells and incubated for 2 h. After washing the cells with staining buffer, PE-conjugated anti-human IgG secondary antibody (Jackson ImmunoResearch, 109-116-098) was applied to the cells. The binding signals were evaluated by flow cytometry using the MACSQuant Analyzer 10 (Miltenyi Biotec) to obtain mean fluorescence intensity (MFI) values.

### Cell cytotoxicity assay for TAVO307_MMAF

CDH17-expressing tumor cells at a density of 2000 cells per well were incubated with TAVO307_MMAF for 3 days. The levels of cell cytotoxicity were assessed by the CellTiter-Glo Luminescent Cell Viability Assay kit (Promega, G7571) according to the manufacturer’s instructions.

### Cytotoxicity by TAVO307_MMAE in tumor cell xenograft model

Mice were housed in a specific pathogen-free facility (12:12 light cycle, 22 ± 1°C, 60% humidity) in individual ventilated cages (5 mice/cage) on corn cob bedding with crumpled tissue paper for enrichment. Animals had ad libitum access to irradiated chow and autoclaved water. BALB/c nude mice, female, 6 to 8 weeks old (GemPharmatech Co., Ltd.), were injected subcutaneously in the right flank with 1 × 10^7^ AsPC-1 or 5 × 10^6^ Ls174T cells (ATCC, CRL-1682, CL-188). CB-17 SCID mice, female, 6 to 8 weeks old (Beijing Vital River Laboratory Animal Technology Co., Ltd.), were injected subcutaneously in the right flank with 5 × 10^6^ SNU-5 cells (ATCC, CRL-5973). All tumor cells were resuspended in 200 μl of a 1:1 ratio of Matrigel (Corning, 354234) to PBS for the establishment of tumor xenografts. When the mean tumor size reached approximately 100–200 mm^3^, all animals were randomly allocated to study groups with 5 mice in each group. The testing ADCs were intravenously administered to the mice at 3 mg/kg on Days 0, 3, 7, and 10 for the SNU-5 model; Days 0 and 7 for the AsPC-1 model; and Days 0 and 3 for the Ls174T model. The dose levels were selected based on prior in-house dose–response studies showing efficacy and tolerability. The primary outcome was tumor volume reduction. Tumor dimensions were assessed using calipers, and the tumor volume was calculated by 1/2 × length × width^2^. Tumor growth inhibition was calculated as the percent reduction in tumor volume in the treated group relative to the control group. For humane endpoints, mice were euthanized if body weight loss was >20% or tumor volume exceeded 3000 mm^3^. Euthanasia was performed by the exposure of mice to a gradually rising concentration of compressed carbon dioxide (CO₂) in a dedicated chamber, delivered at a flow rate displacing 30%–70% of the chamber volume per minute. Daily monitoring included weight, tumor size, and activity. All procedures involving animal care, handling, and treatment complied with the guidelines of the Institutional Animal Care and Use Committee (IACUC) of Suzhou Charles River Accelerator and Development Lab (Approved Protocol: P202302160002-20240705, P202302160002-20240910, P202302160002-20241010). Animals were handled by trained personnel to minimize distress.

### γδ TCR binding assay

Gene fragments encoding the variable and constant domains of Vγ9 (UniProt: Q99603), Vγ4 (UniProt: A0A0C4DH28), Vδ1 (UniProt: A0A1B0GX56), and Vδ2 (UniProt: A0GD36) γδ TCR chains as Fc-fusions were synthesized by IDT and cloned into the pcDNA3.4 expression vector (ThermoFisher, A14697). The Vγ9 or Vγ4 chain construct paired with the Vδ1 or Vδ2 chain construct were co-transfected into Expi293F cells by the Expifectamine293 transfection kit (ThermoFisher, A14525) and correctly-paired heterodimeric γδ TCR Fc fusions were dominantly expressed [49]. Cells were incubated for 5 days at 37°C and the supernatant was harvested and purified by affinity chromatography over a MabSelect SuRe column followed by a desalting column. Protein concentration was determined by Nanodrop with UV absorbance at 280 nm.

The recombinant Vγ9Vδ1TCR, Vγ9Vδ2TCR, Vγ4Vδ1TCR, or Vγ4Vδ2TCR as Fc fusion proteins were coated at the 1 μg/ml concentration in DPBS pH 7.2 on a Maxisorp 96-well plate. After blocking, serial dilutions of test antibodies were applied to the plate and incubated for 2 h at room temperature by shaking. After washing the plates four times with washing buffer, HRP-conjugated anti-human IgG Fab secondary antibody (Jackson ImmunoResearch, 109-036-097) was added for the detection. The plate was washed four times with wash buffer, then TMB substrate was added to the plate for color development. Optical densities were determined with the SpectraMax i3X plate reader at 450 nm wavelength.

### γδ T cell binding assay

For Vγ9Vδ2 T cell expansion, donor PBMCs (peripheral blood mononuclear cells) were seeded in a 6-well flat-bottomed plate at a density of 3 × 10^6^ cells/ml in TexMACS Medium (Miltenyi Biotec, 130-097-196). Zoledronic acid (ZOL, Sigma, SML0223) at 5 μM, recombinant human IL-2 (ProtTech, PT-CF-hIL2) at 100 IU/ml, and recombinant human TGFβ1 (ProtTech, HZ-1011) at 5 ng/ml were added into the culture medium to stimulate the expansion of Vγ9Vδ2 T cells. Two days later, the culture media were supplied with IL-2 at 100 IU/ml and TGFβ1 at 5 ng/ml, and this was repeated every second day for 14 days. At Day 14, the purity of the Vγ9Vδ2 T cell population was assessed by flow cytometry using Alexa Fluor 488 conjugated anti-human CD3 antibody (Biolegend, 300415), PerCP-Vio 700 anti-human TCRγ/δ antibody (Miltenyi Biotec, 130-113-506), and PE anti-Human Vδ2 TCR antibody (BD Biosciences, 555739). The percentage of Vγ9Vδ2 T cells in the whole population was at least 90%.

For Vδ1 T cell expansion, pre-screened donor PBMCs in which the Vδ1 T cells were the dominant γδ T cell population were seeded at a density of 1 × 10^6^ cells/ml in a 24-well flat-bottomed plate pre-coated with 2 μg/ml of an agonistic anti- γδTCR antibody 5A6.E9 (ThermoFisher, TCR1061) with the sushi domain and IL-15 fusion. The culture media was supplemented with IL-2 at 100 IU/ml every other day. On Day 8 after the initial stimulation, the cells were passaged in fresh media and placed in a newly coated 24-well plate with the same agonist to do re-stimulation for another 7 days. At Day 14, the purity of the Vδ1 T cell population was assessed by flow cytometry using Alexa Fluor 488 anti-human CD3 antibody (Biolegend, 300415), PerCP-Vio 700 anti-human TCRγ/δ antibody (Miltenyi Biotec, 130-113-506), and VioBlue anti-human TCR Vδ1 antibody (Miltenyi Biotec, 130-120-583). The percentage of δ1 T cells in the whole population could reach at least 80%.

Bispecific antibody binding to Vδ1 or Vδ2 T cells were evaluated by flow cytometry-based assays. Serial dilutions of antibody were applied to the cells and incubated for 2 h. After washing the cells with staining buffer, PE-conjugated anti-human IgG secondary antibody (Jackson ImmunoResearch, 109-116-098) was applied to the cells. For the Vδ1 T cell binding assay, the small population of Vδ2 T cells was excluded by Brilliant Violet 711 conjugated anti-human TCR Vδ2 antibody (Biolegend, 331412). The binding signals were evaluated by flow cytometry using MACSQuant Analyzer 10 to obtain MFI values.

### γδ TCR-mediated Jurkat cell reporter assay

Gene fragments encoding the open-reading frames of Vγ9, Vδ1, and Vδ2 γδ TCR chains were synthesized by IDT. The Vγ9 was cloned into pSELECT-blasti, and Vδ1 and Vδ2 were cloned into pSELECT-puro vectors (InvivoGen, psetb-mcs, psetp-mcs). The Vγ9 construct paired with either the Vδ1 or Vδ2 construct was co-transfected into the NFAT luciferase reporter Jurkat cell line with the expression of TCR αβ/CD3 complex (BPS Bioscience, 60621) by the Neon electroporation system (ThermoFisher). Jurkat reporter cell lines stably expressing either Vγ9Vδ1TCR or Vγ9Vδ2TCR were obtained by blasticidin and puromycin double selections.

The γδ TCR-mediated Jurkat cell reporter assays were set up in two different formats, either culturing 5 × 10^5^ reporter cells on plates pre-coated with γδ TCE being tested the day before or co-culturing the reporter cells and CDH17-expressing tumor cells (2:1 ratio) along with γδ TCE. The following day, the activation of luciferase reporter gene expression in Jurkat reporter cells was quantified by the ONE-Glo EX Luciferase Assay System (Promega, E8130).

### γδ T cell activation, degranulation, and cytotoxicity assays

The γδ T cell activation assays were set up by culturing expanded Vγ9Vδ2 T cells on 96-well plates pre-coated with TCE being tested the day before. After overnight incubation at 37°C, cells were stained with Alexa Fluor 488 anti-human CD3 antibody (Biolegend, 300415), PerCP-Vio 700 anti-human TCRγ/δ antibody (Miltenyi Biotec, 130-113-506), Super Bright anti-human CD25 antibody (ThermoFisher, 62–0259-42), and APC anti-human CD69 antibody (Biolegend, 310910). The live cells were distinguished by the Zombie R718 fixable viability kit (Biolegend, 423116). For intracellular staining, cells were incubated in pre-coated wells in the presence of GolgiStop containing Monensin (BD Biosciences, 554724) at 37°C overnight. APC anti-human IFN-γ (Biolegend, 502512) was added after cell fixation and permeabilization with Cytofix/Cytoperm Fixation/Permeabilization Kit (BD Biosciences, 554724). Flow cytometry was performed by an Attune NxT cytometer with Cytkick Max (ThermoFisher), and data were analyzed using FlowJo software (BD Biosciences).

The γδ T cell degranulation assay was set up by co-culturing 1 × 10^5^ expanded Vδ1 or Vδ2 T cells and 2 × 10^5^ CDH17-expressing tumor cells (1:2 E:T ratio) along with γδ TCE in the presence of Golgistop (BD Biosciences, 554724) and APC anti-human CD107a antibody (Biolegend, 328620) on 96-well plates. After overnight incubation at 37°C, the γδ T cell degranulation was evaluated by the assessment of CD107a expression on the surface of γδ T cells by flow cytometry.

The γδ T cell cytotoxicity assay was set up by co-culturing 2 × 10^5^ expanded Vδ1 or Vδ2 T cells and 1 × 10^5^ CDH17-expressing tumor cells (2:1 E:T ratio) along with γδ TCE on 96-well plates. The tumor cells were distinguished by staining with eFluor 450 dye (ThermoFisher, 65-0842-90). After overnight incubation at 37°C, the dead and apoptotic tumor cells were characterized by double staining with Apotracker Green (Biolegend, 427403) and DRAQ7 (Abcam, 109202) using flow cytometry. Tumor cell lysis was calculated by subtracting the background percentage of dead and apoptotic cells from the percentage observed in the TCE treated co-culture group. The background cell death was determined from the control groups in which the tumor cells and γδ T cells were co-cultured without antibody treatment.

### PBMC assays for T cells and NK cells

Donor PBMCs labeled with 5 μM Cell Proliferation Dye eFluor 450 (ThermoFisher, 65–0842-90) were mixed with the γδ TCE being tested on 96-well plates. After 3-day incubation at 37°C, cells were stained with Spark Violet 538 anti-human CD45 antibody (Biolegend, 304082), Alexa Fluor 488 anti-human CD3 antibody (Biolegend, 300415), PerCP-Vio 700 anti-human TCRγ/δ antibody (Miltenyi Biotec, 130-113-506), Brilliant Violet 711 anti-human CD56 antibody (ThermoFisher, 407–0566-42), PE anti-human CD25 antibody (Biolegend, 302606), and APC anti-human CD69 antibody (Biolegend, 310910). The live cells were distinguished by the Zombie R718 fixable viability kit (Biolegend, 423116). Flow cytometry was performed by an Attune NxT cytometer with Cytkick Max, and data were analyzed using FlowJo software (BD Biosciences). Representative two-parameter density flow cytometry plots of PBMCs showed the gating strategy to define αβ T cells, γδ T cells, and NK cells (Supplementary Fig. S1). The fractions of proliferated positive cells stained with serially diluted eFluor 450 dyes were quantitated.

### IL-15 functional assays

A HEK-Blue IL-15 reporter assay was conducted with HEK-Blue CD122/CD132 cells (InvivoGen, hkb-il2bg) expressing the IL-15 heterodimeric receptor complexes composed of CD122 and CD132 and the secreted embryonic alkaline phosphatase (SEAP) reporter. 5 × 10^4^ cells seeded in the 96-well plates were treated with serial dilutions of testing antibodies. After overnight incubation, the SEAP activity was quantitated by adding Quanti-Blue solution (InvivoGen, rep-qbs).

A CTLL-2 cell proliferation assay was conducted by seeding 20 000 CTLL-2 cells (ATCC, TIB-214) in a 96-well plate in RPMI-1640 complete growth medium and treated them with serial dilutions of testing antibodies. Three days after treatment, CTLL-2 cell proliferation was quantitated using a CellTiter-Glo luminescent cell viability assay kit (Promega, G7571).

### Statistical analysis

The assay data or normalized data were plotted against the logarithm of test article concentrations. Four-parameter logistic sigmoidal dose–response analyses were performed by GraphPad Prism 10.2.3 (GraphPad Software) to calculate the EC_50_ values.

## Results

### Target binding by a humanized anti-CDH17 antibody TAVO307

**Figure 1:**
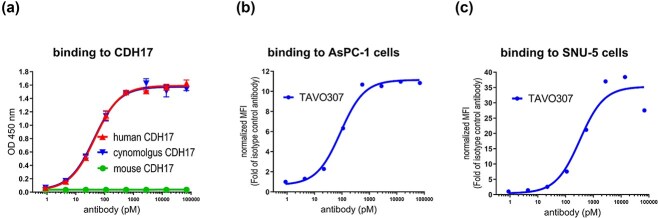
Binding properties of the anti-CDH17 antibody TAVO307. (a) The ELISA binding assays showed TAVO307 antibody binding to human, cynomolgus monkey, and mouse CDH17 proteins. The *y*-axis absorbance at 450 nm was plotted against the *x*-axis concentrations of test antibodies. Experiments were performed in duplicate with data reported as mean ± SD. (b and c) Flow cytometry-based binding assays showed TAVO307 antibody binding to AsPC-1 (b) and SNU-5 cells (c). The *y*-axes MFI values normalized to the MFI value of an isotype control antibody were plotted against the *x*-axes test antibody concentrations. Data shown were from a single experiment.

A humanized monoclonal anti-CDH17 antibody, named TAVO307, was evaluated for its binding to recombinant human, cynomolgus monkey, and mouse CDH17 coated on plates by ELISA-based binding assays. The antibody showed potent binding to human CDH17 with an EC_50_ of 46 pM. TAVO307 also bound with similar potency to cynomolgus monkey CDH17 (EC_50_ = 44 pM) but did not recognize murine CDH17 (Fig. 1a). By flow cytometry assays, TAVO307 bound potently to CDH17 expressed on AsPC-1 and SNU-5 cells with EC_50_ of 89 and 328 pM, respectively (Fig. 1b and c).

### Cytotoxicity of CDH17-expressing GI cancer cells by TAVO307_MMAF

To explore whether CDH17 can be employed as a tumor-associated antigen to mediate the cytotoxicity of GI cancers, CDH17 antibody–drug conjugates (ADCs) were made to guide the cytotoxic payload into CDH17-expressing cells. Relative to the same-class payload MMAE, MMAF is less cell-permeable and hence cause less bystander killing effect. For the better demonstration of CDH17-targeting effects, we conjugated MMAF to the anti-CDH17 antibody (TAVO307_MMAF). By SDS-PAGE under the reduced condition, the heavy chain band of TAVO307_MMAF showed a higher shift in molecular weight relative to the unconjugated TAVO307 heavy chain due to the conjugation of two MMAF cytotoxic drug molecules per antibody (DAR = 2), while the molecular weights of light chains were unchanged (Supplementary Fig. S2a).

**Figure 2:**
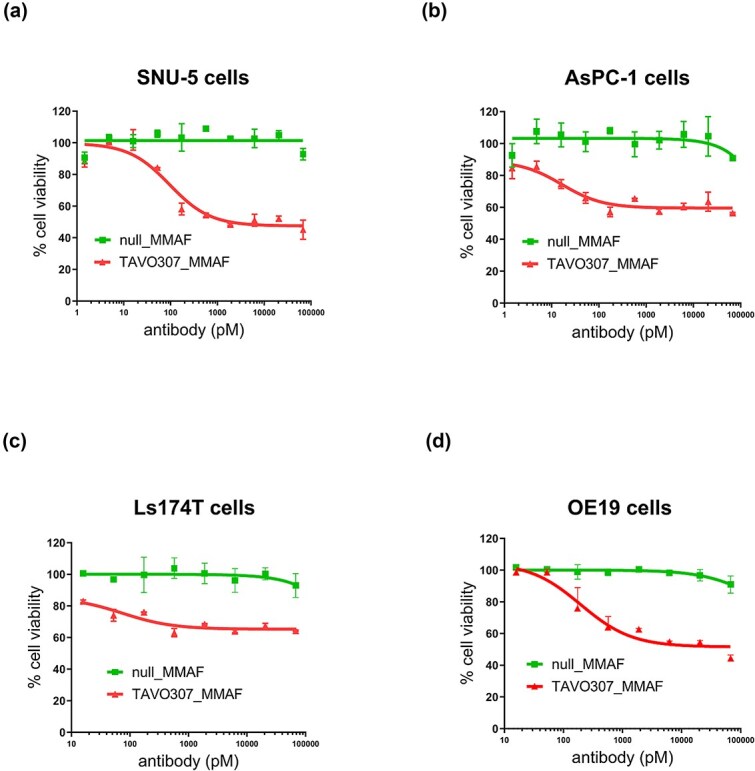
Cytotoxicity of CDH17-expressing tumor cells by the TAVO307_MMAF. The cytotoxicity assays showed the concentration-dependent cytotoxicity of SNU-5 (a), AsPC-1 (b), Ls174T (c), and OE19 cells (d) by the MMAF conjugated anti-CDH17 (TAVO307_MMAF) and null control (null_MMAF) antibodies. The *y*-axes percentages of cell viability relative to the case without antibody added were plotted against the *x*-axes concentrations of the respective test articles. Experiments were performed in duplicate with data reported as mean ± SD.

The cytotoxicity of CDH17-expressing tumor cells by TAVO307_MMAF was assessed by cell cytotoxicity assays. The TAVO307_MMAF showed concentration-dependent cytotoxicity to CDH17-expressing gastric cancer cell SNU-5 (EC_50_ = 95 pM), pancreatic cancer cell AsPC-1 (EC_50_ = 16.5 pM), colon cancer cell Ls174T (EC_50_ = 74.6 pM), and esophageal cancer cell OE19 (EC_50_ = 197 pM) (Fig. 2), while a null antibody conjugated with MMAF (null_MMAF) showed minimal cell cytotoxicity.

### Efficacy of TAVO307_MMAE in mouse tumor xenograft models

The CDH17-ADC mediated cytotoxicity of GI cancer cells were also evaluated in mouse tumor xenograft models. We chose MMAE as the payload to capture the aggregated effects of CDH17-based payload targeting and bystander killing in tumor elimination. TAVO307 was conjugated with MMAE by cysteine-based cytotoxic drug conjugation. Multiple chromatographic peaks corresponding to antibodies conjugated with different numbers of MMAE were observed by HIC, and the average DAR for TAVO307_MMAE was estimated as 3.6 (Supplementary Fig. S2b). In SEC, TAVO307_MMAE migrated as a single monodisperse chromatographic peak without minor peaks that were indicative of protein aggregation or degradation (Supplementary Fig. S2c).

**Figure 3:**
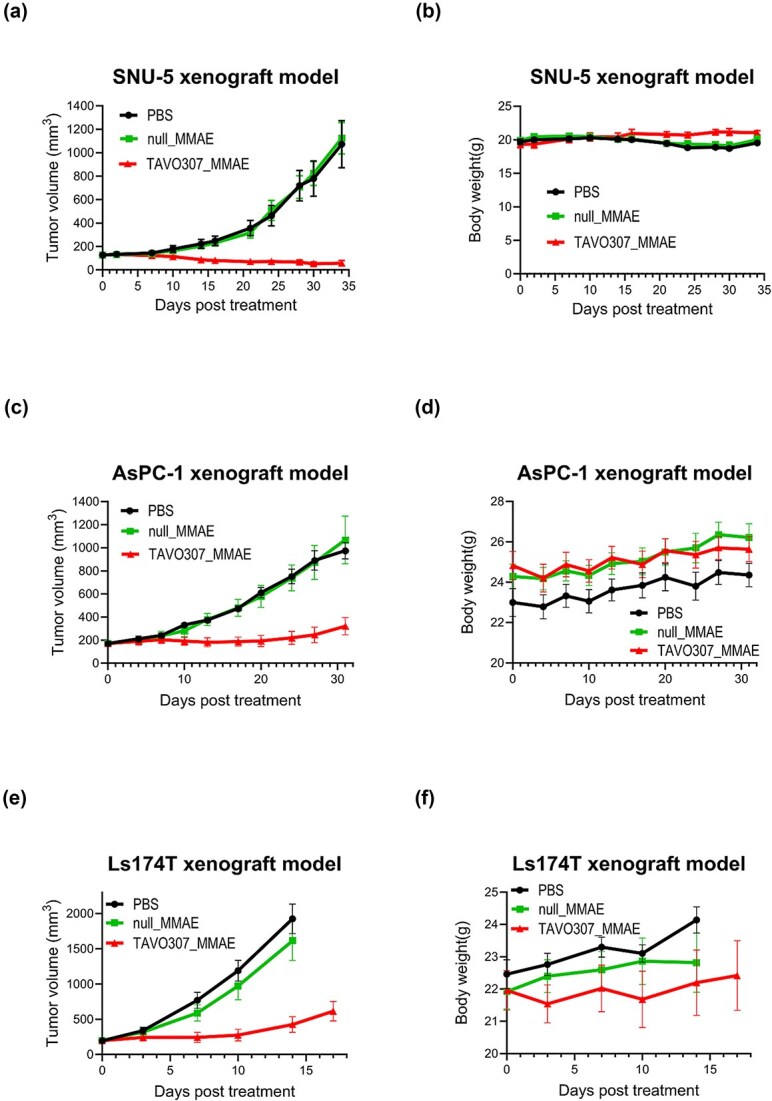
Efficacies of TAVO307_MMAE in CDH17-expressing tumor cell xenograft models. Xenograft tumor models showed the tumor growth inhibition efficacy of TAVO307_MMAE and null_MMAE dosed at 3 mg/kg and PBS control in SNU-5 (a), AsPC-1 (c), and Ls174T (e) cell xenograft models. The *y*-axes tumor volumes (mm^3^) were plotted against the *x*-axes days post treatment (the data expressed as mean ± SEM values, *n* = 5). Figures (b, d, and f) showed the corresponding mice body weights upon the test article treatments. The *y*-axes body weights were plotted against the *x*-axes days post treatment (the data expressed as the mean ± SEM values, *n* = 5).

The efficacies of the TAVO307_MMAE in tumor cell killing were evaluated in several GI cancer xenograft models. In the gastric cancer SNU-5 model, TAVO307_MMAE dosed at 3 mg/kg twice per week for a total of 4 times led to significant tumor shrinkage with 95% tumor growth inhibition at Day 34 compared to untreated mice or mice treated with a null antibody conjugated with MMAE (Fig. 3a). One out of five mice treated with TAVO307_MMAE achieved complete tumor remission. In the pancreatic cancer AsPC-1 model, TAVO307_MMAE dosed weekly at 3 mg/kg for two weeks also led to significant tumor shrinkage with 67% tumor growth inhibition at Day 31 (Fig. 3c). In the colon cancer Ls174T model, TAVO307_MMAE dosed at 3 mg/kg at Day 0 and Day 3 led to 78% tumor growth inhibition at Day 14 (Fig. 3e). In all these xenograft models, mice maintained normal body weights throughout the treatment period (Fig. 3b, d, and f).

### Identification and characterization of a VHH antibody binding both Vδ1 and Vδ2 types of γδ T cell receptors

Besides CDH17-based ADC, we also explored whether CDH17 could be employed as a tumor-associated antigen to guide a TCE to bridge T cells to kill GI cancer cells. Instead of classical αβ T cells, we developed a TCE to engage γδ T cells for tumor killing. For this, we immunized Alpaca and screened for single domain VHH antibodies that recognized human γδ TCR. More specifically, Vγ4Vδ1 and Vγ4Vδ2 TCR as Fc fusion proteins were used as immunogens, followed by positive selections on Vγ9Vδ1 and Vγ9Vδ2 TCR as Fc fusion proteins and counter selection against IgG1 Fc. The binding selectivity of identified hits were evaluated by ELISA-based binding assays to different types of γδ TCR as Fc fusion proteins, as well as flow cytometry based binding assays and functional reporter assays on Jurkat NFAT reporter cell lines stably expressing either Vγ9Vδ1 or Vγ9Vδ2 TCR. One of the hits, designated as VHH371, specifically bound to all subtypes of γδ TCR. By the knob-in-hole technology, we made a bispecific antibody as a CDH17-based γδ TCE, TAVO307 × VHH371, with the TAVO307 Fab arm for CDH17 binding, the VHH371 arm for γδ TCR binding, and an IgG1 Fc with L234A and L235A mutations to reduce Fc effector functions (Supplementary Fig. S3a). In SEC, TAVO307 × VHH371 migrated as a major single chromatographic peak with insignificant minor peaks as signs for protein aggregation and degradation (Supplementary Fig. S3b).

**Figure 4:**
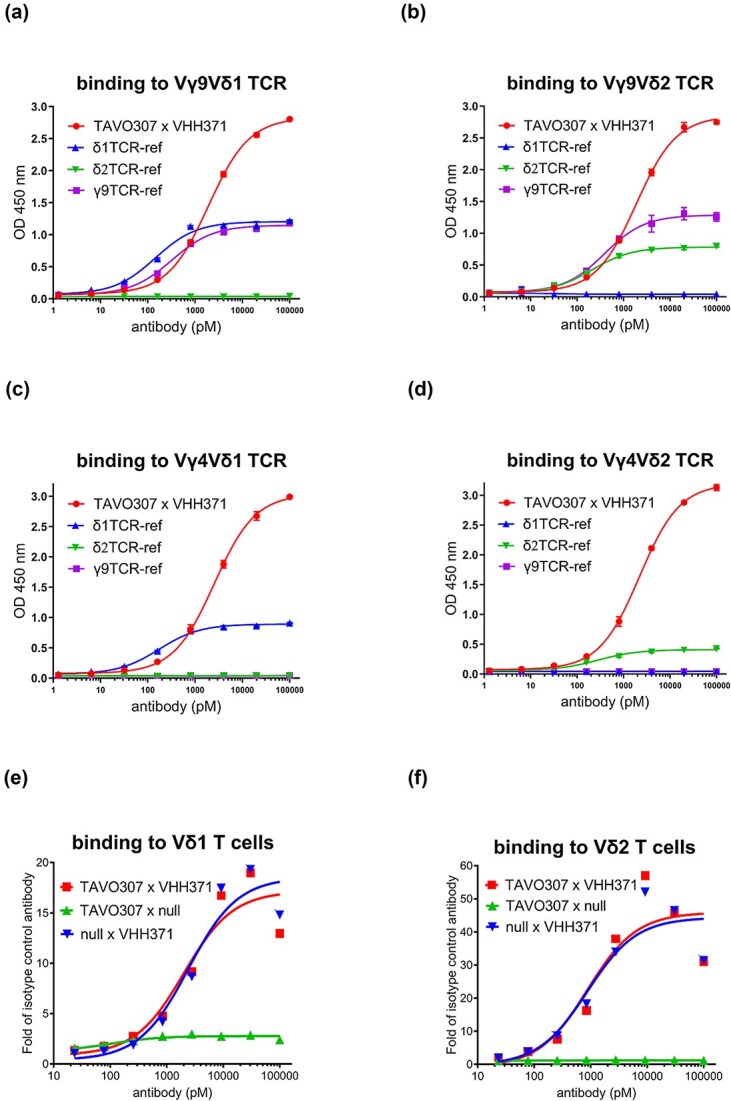
VHH371 binding to all subtypes of γδ TCR. (a–d). ELISA binding assays showed TAVO307 × VHH371, δ1TCR-ref, δ2TCR-ref, and γ9TCR-ref antibodies binding to recombinant human Vγ9Vδ1 (a), Vγ9Vδ2 (b), Vγ4Vδ1 (c), and Vγ4Vδ2 (d) subtypes of γδ TCR, respectively. The *y*-axes absorbances at 450 nm were plotted against the *x*-axes concentrations of the respective antibodies. Experiments were performed in duplicate with data reported as mean ± SD. (e and f) Flow cytometry-based cell binding assays showed TAVO307 × VHH371, TAVO307 × null, and null × VHH371 bispecific antibodies binding to the Vδ1 (e) and Vδ2 (f) T cells. The *y*-axes MFI folds over isotype control antibody were plotted against the *x*-axes concentrations of the test antibodies. Data shown are from a single experiment.

TAVO307 × VHH371 binding to recombinant γδ T cell receptors was confirmed by ELISA-based binding assays. In these assays, recombinant human Vγ9Vδ1, Vγ9Vδ2, Vγ4Vδ1, Vγ4Vδ2 TCR as Fc fusion proteins were coated on ELISA plates. The differential bindings to reference antibodies specific to Vδ1, Vδ2, and Vγ9 subtypes of γδ TCR validated the subtype specificities of these antigens. TAVO307 × VHH371 concentration-dependently bound to all subtypes of γδ TCR antigens with EC_50_ of 1.92 nM for Vγ9Vδ1, 1.87 nM for Vγ9Vδ2, 2.54 nM for Vγ4Vδ1, and 2.20 nM for Vγ4Vδ2 (Figs. 4a–d), indicating that VHH371 is a pan-γδ TCR binder. Besides, VHH371-containing bispecific antibodies, either paired with a CDH17 antibody arm (TAVO307 × VHH371) or a null antibody arm (null × VHH371), concentration-dependently bound to both Vδ1 and Vδ2 T cells expanded from donor PBMC with EC_50_ of 2.0 and 0.9 nM for TAVO307 × VHH371 binding to Vδ1 and Vδ2 T cells respectively (Fig. 4e and f), while a bispecific antibody lacking the VHH371 arm (TAVO307 × null) failed to bind to γδ T cells in flow-cytometry-based cell binding assays. On the other hand, TAVO307 × VHH371 and TAVO307 × null bispecific antibodies bound to AsPC-1 cells with similar profiles (EC_50_ ~ 0.2 nM, Supplementary Fig. S3e), while the null × VHH371 antibody did not bind to the CDH17-expressing cells.

### VHH371 mediated the activation of both Vδ1 and Vδ2 types of γδ T cell receptors

**Figure 5:**
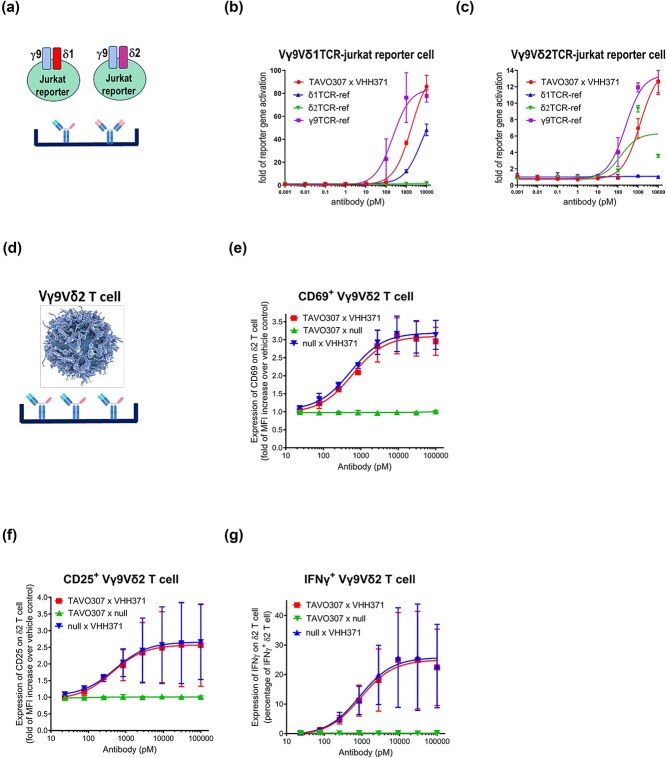
VHH371 activation of the γδ TCR. (a). The cartoon diagram showed the reporter assays with antibodies immobilized on the plate activating Jurkat reporter cells expressing Vγ9Vδ1TCR or Vγ9Vδ2TCR. (b and c) Jurkat cell NFAT reporter assays showed immobilized TAVO307 × VHH371, δ1TCR-ref, δ2TCR-ref, and γ9TCR-ref antibodies activating luciferase reporter gene expression in Jurkat NFAT reporter cells expressing Vγ9Vδ1TCR (b) or Vγ9Vδ2TCR (c). The *y*-axes folds of reporter gene activation over vehicle control were plotted against the *x*-axes concentrations of the respective antibodies. Experiments were performed in duplicate with data reported as mean ± SD. (d) The cartoon diagram showed the γδ T cell activation assays with antibodies immobilized on the plate activating Vγ9Vδ2 T cells. (e–g) γδ T cell activation assays showed immobilized TAVO307 × VHH371, TAVO307 × null, and null × VHH371 antibodies activating the Vγ9Vδ2 T cell expression of CD69 (e), CD25 (f), and IFNγ (g). The *y*-axes folds of MFI increase over vehicle control (e and f) or the percentage of IFNγ-positive δ2 T cells (g) were plotted against the *x*-axes concentrations of the respective antibodies. Experiments were performed in duplicate with data reported as mean ± SD.

The functional activities of VHH371 in activating γδ T cell receptors were assessed by Jurkat cell NFAT reporter assays, in which testing antibodies precoated on plates were incubated with Jurkat NFAT reporter cell lines stably expressing either Vγ9Vδ1 or Vγ9Vδ2 TCR overnight (Fig. 5a). The luciferase reporter gene in cells expressing Vγ9Vδ1TCR was activated by δ1TCR-ref and γ9TCR-ref antibodies but not by the δ2TCR-ref antibody, confirming the expected subtype-specific activation of the Vγ9Vδ1TCR-expressing reporter cells (Fig. 5b). Similarly, the subtype-specific activation of the Vγ9Vδ2TCR-expressing reporter cells was validated by its activation by δ2TCR-ref and γ9TCR-ref antibodies but not by δ1TCR-ref antibody (Fig. 5c). The TAVO307 × VHH371 bispecific antibody, when coated, concentration-dependently activated reporter gene expression from Jurkat reporter cells expressing either Vγ9Vδ1TCR or Vγ9Vδ2TCR (Fig. 5b and c), confirming VHH371 as a pan-γδ TCR agonist antibody.

The functional activity of VHH371 was also evaluated in a γδ T cell activation assay. In this assay, testing antibodies coated on cell plates were incubated with Vγ9Vδ2 T cells expanded from donor PBMC overnight, and the CD25, CD69, and IFN-γ expressions on Vγ9Vδ2 T cells were quantitated (Fig. 5d). VHH371-containing bispecific antibodies, TAVO307 × VHH371 and null × VHH371, concentration-dependently activated CD25, CD69, and IFN-γ expressions on Vγ9Vδ2 T cells (Fig. 5e–g), while TAVO307 × null failed to activate Vγ9Vδ2 T cells.

### TAVO307 × VHH371 mediated the activation of γδ TCR reporter cells by CDH17-expressing tumor cells

**Figure 6:**
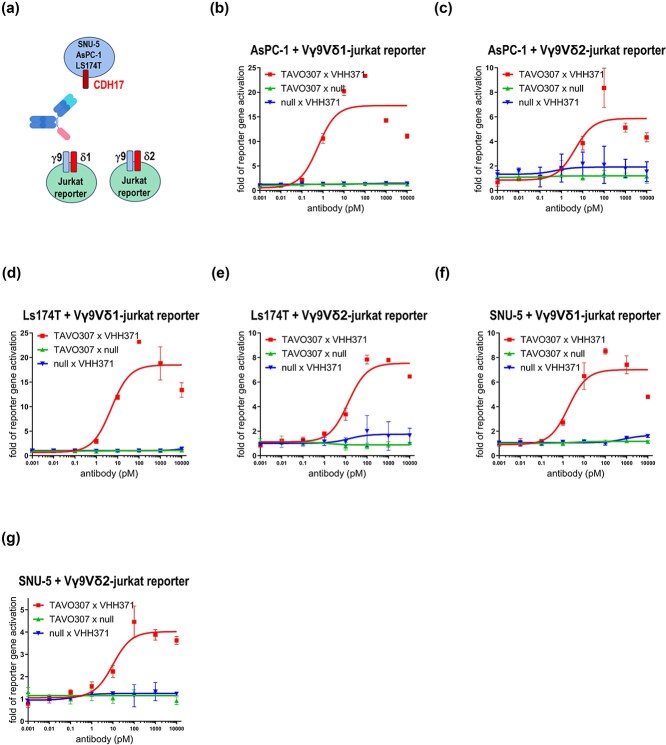
TAVO307 × VHH371 mediated the activation of γδ TCR Jurkat reporter cells with CDH17-expressing tumor cells. (a) The cartoon diagram showed the reporter assays with γδ T cell engagers mediating the activation of Jurkat reporter cells expressing Vγ9Vδ1TCR or Vγ9Vδ2TCR by CDH17-expressing tumor cells. In these assays, reporter cells were co-cultured with CDH17-expressing tumor cells (2:1 ratio) for 1 day then the luciferase reporter activities were quantitated. (b–g) Jurkat cell NFAT reporter assays showed TAVO307 × VHH371, TAVO307 × null, null × VHH371 bispecific antibodies in mediating AsPC-1 (b and c), LS174T (d and e), and SNU-5 (f and g) cells in activating reporter gene expression in Jurkat NFAT reporter cells expressing either Vγ9Vδ1TCR (b, d, and f) or Vγ9Vδ2TCR (c, e, and g). The *y*-axes folds of reporter gene activation over vehicle control were plotted against the *x*-axes concentrations of the respective antibodies. Experiments were performed in duplicate with data reported as mean ± SD.

The functional activity of TAVO307 × VHH371 in mediating the activation of γδ T cell receptors by CDH17-expressing tumor cells was assessed by Jurkat cell NFAT reporter assays, in which testing antibodies were incubated with Jurkat NFAT reporter cells stably expressing either Vγ9Vδ1TCR or Vγ9Vδ2TCR co-cultured with CDH17-expressing tumor cells (Fig. 6a). In the presence of AsPC-1 cells, TAVO307 × VHH371 concentration-dependently facilitated reporter gene expression from Jurkat reporter cells expressing either Vγ9Vδ1TCR or Vγ9Vδ2TCR with EC_50_ of 0.6 and 4.4 pM, respectively (Fig. 6b and c), while the null control antibodies, either lacking the CDH17-binding arm (null × VHH371) or lacking the γδ TCR-binding arm (TAVO307 × null), failed to mediate reporter gene expressions. Similarly, TAVO307 × VHH371 antibody also mediated LS174T and SNU-5 cells in the activation of Vγ9Vδ1TCR or Vγ9Vδ2TCR expressed from the Jurkat reporter cells and led to reporter gene activation (Fig. 6d–g).

### TAVO307 × VHH371 mediated the cytotoxic degranulation of γδ T cells by CDH17-expressing tumor cells

**Figure 7:**
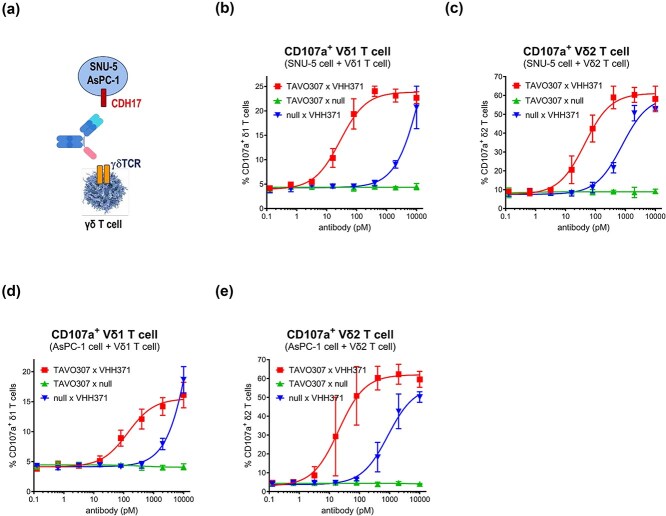
TAVO307 × VHH371 mediated γδ T cell degranulation upon ligation to CDH17-expressing tumor cells. (a) The cartoon diagram showed γδ T cell engagers mediating γδ T cell degranulation and cytotoxicity of CDH17-expressing tumor cells. (b–e) For γδ T cell degranulation assays, testing antibodies were added to 1 × 10^5^ Vδ1 or Vδ2 T cells (expanded from PBMC obtained from a single donor) co-cultured with 2 × 10^5^ CDH17-expressing tumor cells (1:2 E:T ratio). After 4 h incubation, the expression of CD107a on γδ T cells was quantitated by flow cytometry. TAVO307 × VHH371, TAVO307 × null, null × VHH371 bispecific antibodies were tested for activation of CD107a degranulation marker expression in Vδ1 (b and d) or Vδ2 (c and e) T cells by SNU-5 (b and c) and AsPC-1 (d and e) cells. The y-axes percentages of CD107a-positive γδ T cells were plotted against the *x*-axes concentrations of the respective antibodies. Experiments were performed in duplicate or triplicate with data reported as mean ± SD.

The cytotoxic degranulation of Vδ1 T cells and Vδ2 T cells by TAVO307 × VHH371 bispecific antibody upon co-culturing with CDH17-expressing cells was evaluated in γδ T cell degranulation assays. In these assays, testing antibodies were added to 1 × 10^5^ Vδ1 or Vδ2 T cells co-cultured with 2 × 10^5^ CDH17-expressing tumor cells (1:2 E:T ratio). After 4 h incubation, the expression of CD107a on γδ T cells, which was the hallmark of T cell cytotoxic degranulation, was quantitated by flow cytometry (Fig. 7a). In the presence of SNU-5 cells, TAVO307 × VHH371 concentration-dependently facilitated the up-regulation of the CD107a marker on either Vδ1 or Vδ2 T cells with EC_50_ of 28.2 and 43.8 pM, respectively (Fig. 7b and c). The null control antibody lacking the CDH17-binding arm, null × VHH371, stimulated CD107a expression on Vδ1 or Vδ2 T cells with much less potency, while TAVO307 × null failed to mediate CD107a expression. Similarly, TAVO307 × VHH371 antibody also mediated AsPC-1 cells in the activation of T cell degranulation marker CD107a expression on Vδ1 or Vδ2 T cells with EC_50_ of 137.8 and 20.4 pM, respectively (Fig. 7d and e).

### TAVO307 × VHH371 mediated γδ T cells in the cytotoxicity of CDH17-expressing tumor cells

**Figure 8:**
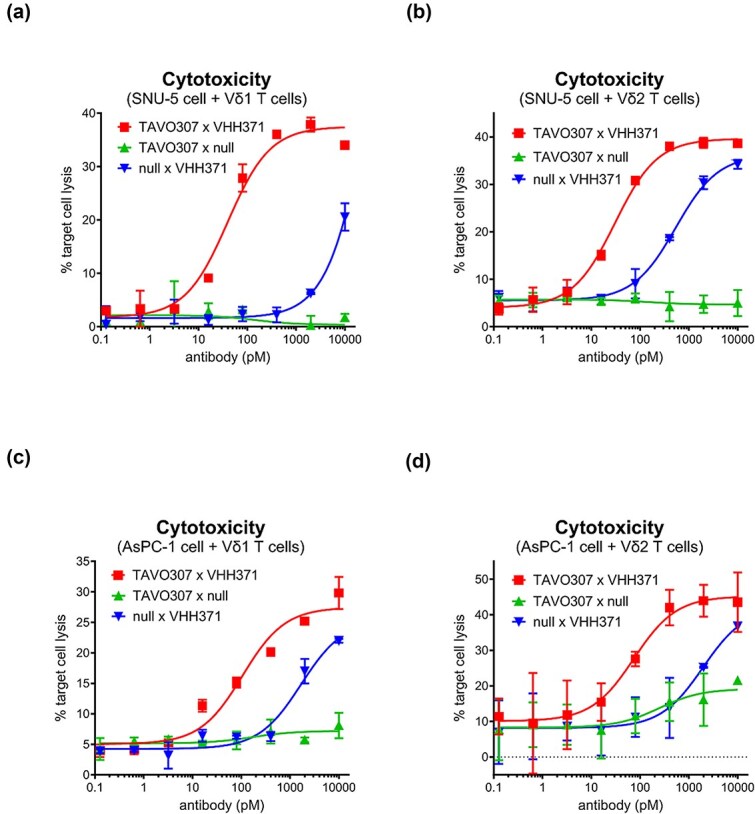
TAVO307 × VHH371 mediated γδ T cell in the cytotoxicity of CDH17-expressing tumor cells. 2 × 10^5^ γδ T cells expanded from PBMCs obtained from a single donor were co-cultured with 1 × 10^5^ CDH17-expressing tumor cells (2:1 E:T ratio) in the presence of serial dilutions of testing antibodies for 24 h. The percentages of target cell lysis were determined by flow cytometry. γδ T cell cytotoxicity assays showed TAVO307 × VHH371, TAVO307 × null, null × VHH371 bispecific antibodies in mediating the cytotoxicity of SNU-5 (a and b) and AsPC-1 (c and d) cells by Vδ1 (a and c), or Vδ2 (b and d) T cells. The *y*-axes percentages of target cell lysis were plotted against the *x*-axes concentrations of the respective antibodies. Experiments were performed in duplicate with data reported as mean ± SD.

The TCE-mediated cytotoxicity of CDH17-expressing tumor cells by γδ T cells was evaluated in γδ T cell cytotoxicity assays. 2 × 10^5^ expanded γδ T cells were co-cultured with 1 × 10^5^ CDH17-expressing tumor cells (2:1 E:T ratio) in the presence of serial dilutions of testing antibodies for 24 h. The percentages of target cell lysis were determined by flow cytometry (Fig. 7a). TAVO307 × VHH371 concentration-dependently facilitated either Vδ1 or Vδ2 T cells in the cytotoxicity of SNU-5 cells with EC_50_ of 39.7 and 30.5 pM, respectively (Fig. 8a and b). The null × VHH371 antibody also mediated some cytotoxicity of SNU-5 cells by Vδ1 or Vδ2 T cells but with much less potency. Likewise, the TAVO307 × null failed to mediate tumor cell killing. Similarly, TAVO307 × VHH371 antibody also mediated the cytotoxicity of AsPC-1 cells by Vδ1 or Vδ2 T cells with EC_50_ of 105.7 and 74.4 pM, respectively (Fig. 8c and d).

### IL-15 engineered on TAVO307 × VHH371 enhanced γδ T cell proliferation and activation

Interleukin-15 (IL-15) is a pleiotropic cytokine crucial for the proliferation and activation of T cells, including both αβ and γδ T cells, and NK cells. To overcome the limited quantity and poor persistence of γδ T cells in tumor cell cytotoxicity, we generated a CDH17-based γδ TCE with IL-15 fusion, TAVO307 × VHH371_IL15, with the sushi domain (SD) of IL-15 receptor α followed by IL-15 as a fusion protein engineered only at the C-terminus of the VHH371 chain (Supplementary Fig. S3c). Considering the toxicity of native IL-15, we chose an attenuated form of IL-15 with S7T and I68K mutations that weakened IL-15 activity 10–100-fold relative to the native one in various IL-15 functional assays (Supplementary Table S1). In SEC, TAVO307 × VHH371_IL15 migrated as a major single chromatographic peak with a minor peak (Supplementary Fig. S3d).

The functional IL-15 activity of TAVO307 × VHH371_IL15 was evaluated in a HEK-Blue IL-15 reporter assay. TAVO307 × VHH371_IL15 activated SEAP reporter expression with an EC_50_ of 11.33 pM, while TAVO307 × VHH371 did not activate the reporter expression (Supplementary Fig. S4a). The IL-15 activity was also confirmed in a CTLL-2 cell proliferation assay. The TAVO307 × VHH371_IL15 stimulated CTLL-2 cell proliferation with an EC_50_ of 214.3 pM, whereas the TAVO307 × VHH371 failed to activate cell proliferation (Supplementary Fig. S4b).

**Figure 9:**
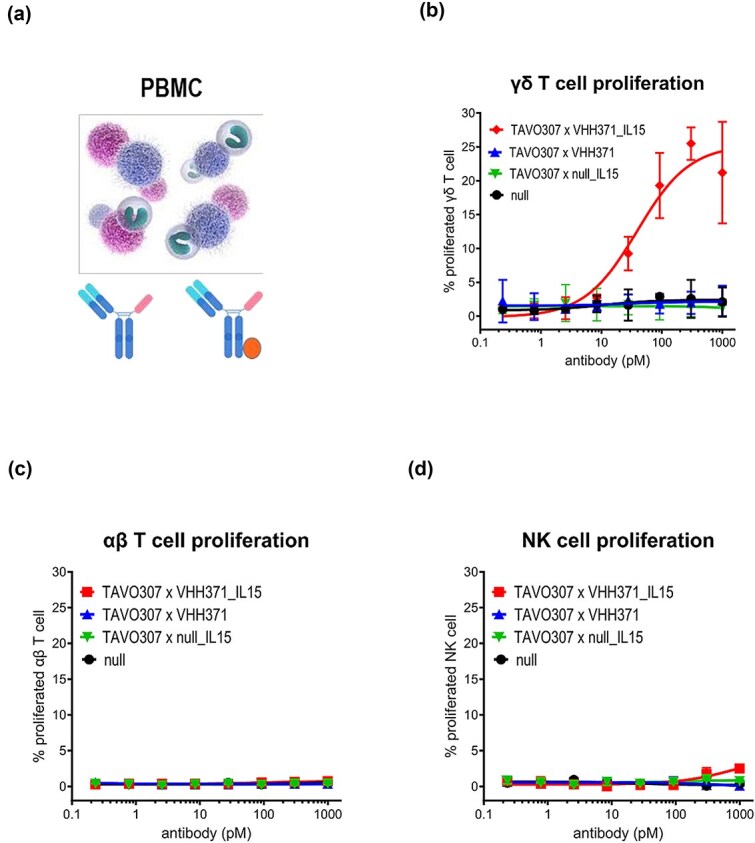
IL-15 fusion facilitated γδ T cell proliferation dependent on γδ TCR binding. (a) The cartoon diagram showed γδ T cell engagers with IL-15 fusion mediating the proliferation and activation of T cells and NK cells in PBMC. (b–d) Cell proliferation assays of TAVO307 × VHH371_IL15, TAVO307 × VHH371, TAVO307 × null_IL15, and null antibodies assessing the proliferation of γδ T cells (b), αβ T cells (c), and NK cells (d). The testing antibodies were incubated with PBMC isolated from a single donor for 3 days. The proliferations of distinct cell populations were quantitated by flow cytometry assays. The *y*-axes percentages of γδ T cell proliferation were plotted against the *x*-axes concentrations of the respective antibodies. Experiments were performed in duplicate with data reported as mean ± SD.

**Figure 10:**
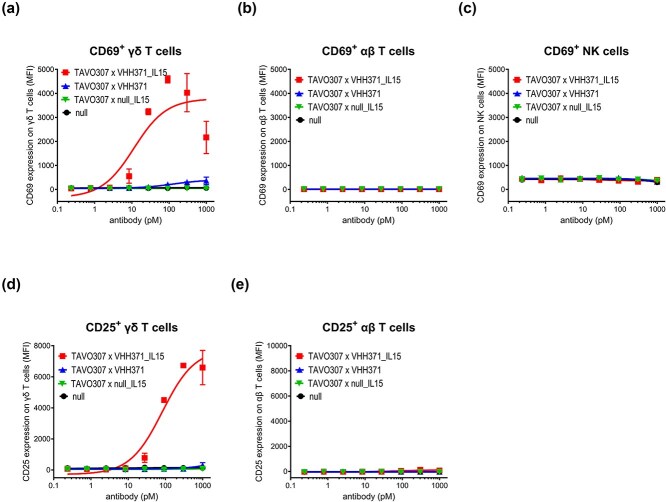
IL-15 fusion facilitated γδ T cell activation dependent on γδ TCR binding. Cell activation assays showed TAVO307 × VHH371_IL15, TAVO307 × VHH371, TAVO307 × null_IL15, and null antibodies mediating the expression of CD69 activation marker on γδ T cells (a), αβ T cells (b), and NK cells (c), and the CD25 activation marker expression on γδ T cells (d), and αβ T cells (e). The testing antibodies were incubated with PBMC isolated from a single donor for 3 days. The activation marker (CD69 and CD25) expressions on distinct cell populations were quantitated by flow cytometry assays. The *y*-axes MFIs representing CD69 or CD25 expression on cells were plotted against the *x*-axes concentrations of the respective antibodies. Experiments were performed in duplicate with data reported as mean ± SD.

The contributions of engineered IL-15 fusion to the proliferation and activation of γδ T cells as well as αβ T cells and NK cells were evaluated by incubating the testing antibodies with donor PBMC for 3 days, followed by the quantitation of cell proliferation and activation markers (CD69 and CD25) on distinct cell populations by flow cytometry assays (Fig. 9a). TAVO307 × VHH371_IL15 concentration dependently activated the proliferation of γδ T cell with EC_50_ of 39.1 pM (Fig. 9b) without activating αβ T or NK cell proliferation (Fig. 9c and d). The proliferation activation was attributed to the engineered IL-15 fusion since the TAVO307 × VHH371 failed to activate γδ T cell proliferation. Besides, the TAVO307 × null_IL15 antibody, in which the VHH371 arm was replaced by a null arm, failed to activate γδ T cell proliferation, indicating the proliferation activation depended on the presence of the γδ T cell binding arm (Fig. 9B). Similarly, TAVO307 × VHH371_IL15 upregulated the expression of activation markers CD69 and CD25 on γδ T cells in the IL-15 fusion and γδ T cell binding dependent manner with EC_50_ of 11.4 and 82.9 pM, respectively (Fig. 10a and d). However, TAVO307 × VHH371_IL15 at these concentrations failed to drive the activation of αβ T cells or NK cells (Fig. 10b, c, and  e). Albeit, these results were obtained from *ex vivo* assays with PBMCs from a single donor.

## Discussion

GI cancers, including colorectal cancer and pancreatic cancer (with the highest mortality rate), account for one-third of cancer-related mortality globally [50]. Only a limited number of GI tumor-associated antigens, which include EGFR, HER2, Claudin18.2, CEACAM5, and B7-H3, are being explored as targets for GI cancers and they suffer from on-target off-tumor toxicity [51]. In addition, multidrug resistance remains a major challenge for GI cancer small molecule treatments [52, 53]. CDH17, with high levels of expression across different classes of GI cancers but with low and restricted normal physiological expression, is becoming a prominent therapeutic target for the development of effective and safe therapeutics for GI cancers. However, anti-CDH17 mAb directed signaling inhibition of tumor progression and metastasis only retards tumor growth but is not sufficient for tumor eradication [12, 15, 54]. In this work, we described a monoclonal humanized anti-CDH17 antibody TAVO307 with picomolar binding affinity and advanced the therapeutic targeting of CDH17 in GI cancers through either the cytotoxic ADC or γδ T cell redirection modality.

In *in vitro* and *in vivo* studies, our CDH17-targeting ADCs demonstrated potent tumor cytotoxicity towards GI cancer cell lines expressing CDH17. These results aligned with prior preclinical data on CDH17-ADCs, reinforcing the rationale for CDH17 targeting in GI malignancies, although more work is needed for payload and DAR selection and optimization. Current targeted therapies, including EGFR and HER2 drugs, claudin 18.2 inhibitors, VEGF blockers, and immune checkpoint inhibitors, have shown limited efficacy in small subgroups of gastric, esophageal, and colorectal cancers [55, 56]. CDH17 ADCs could complement these GI therapies when used in combination to achieve synergistic therapeutic potential. However, our current animal models were limited in capturing the relevant role of CDH17 in promoting tumor progression and metastasis in a clinical setting. Future studies with more sophisticated orthotopic and metastasis cancer models established with transgenic human CDH17 mice could provide better assessments of the efficacy of CDH17-ADCs. PDX model studies could further assess the efficacies of CDH17-ADCs on tumor heterogeneity and payload bystander effects.

The toxicity profile of our CDH17-ADC molecule remains to be established. CDH17 is predominantly localized on the lateral membranes of intestinal epithelium. Although relatively inaccessible to circulating antibodies under normal conditions, the intestinal epithelium may still be vulnerable in the context of barrier damage or inflammation. A detailed toxicology profile for CDH17-ADC molecules could be established in mice transgenic with human CDH17 or nonhuman primates prior to clinical translation. Although several CDH17-ADCs are in clinical trial stages, these trials are still very early, and no significant clinical toxicities have been publicly reported yet. The payloads for these CDH17-ADC molecules employ either MMAE (e.g. TORL-3-600) or topoisomerase-I (e.g. AMT-676, 7MW4911)-based inhibitors. Typical adverse events for MMAE ADCs include peripheral neuropathy and neutropenia, while topoisomerase-I ADCs often show neutropenia and diarrhea. The incidence/severity of toxicity specifically for CDH17 ADCs must be empirically addressed to guide the refinement of linkers and payloads to balance safety and anti-tumor efficacy.

To complement the ADC strategy, we also explored a γδ TCE strategy to harness the unique biology of γδ T cells for tumor cell cytotoxicity. We identified a VHH antibody VHH371 capable of binding both the Vδ1 and Vδ2 types of TCR chains. The variable regions of different subtypes of γδ TCR chains are highly diverse for enabling broad antigen recognition, while the constant regions are highly conserved for maintaining structural integrity, pairing, and signaling competence of the receptor complex. We speculated that VHH371 bound to an epitope in the constant regions conserved among all γδ TCR chains to allow pan-subtype binding. In addition, the VHH371 was able to function as an agonist antibody, driving the activation of both Vδ1 and Vδ2 TCR engineered on Jurkat cells, as well as both Vδ1 and Vδ2 T cells, leading to the up-regulation of T cell activation markers. VHH371 might activate γδ TCR in a way different from the typical γδ T cell antigens that usually bind to the V regions of γδ TCR. It could induce a conformational change of the constant region of γδ TCR that triggers signaling leading to receptor activation, a mechanism reported for a few antibodies with similar properties [57, 58]. Further studies, including epitope mapping, mutational analysis, and structural modeling, are needed to test this hypothesis. Similar to other agonist antibodies, the VHH371 antibody crosslinking could be a prerequisite to cluster multiple γδ TCR for activation, which could have resulted from either coating onto a plate surface or by ligation to tumor cells through the TCE format, while the soluble VHH371 antibody did not show agonism as a monomeric form.

Upon ligation to CDH17-expressing tumor cells, the TAVO307 × VHH371 TCE drove the activation of both Vδ1 and Vδ2 TCR engineered on Jurkat cells in reporter assays. This molecule also activated CD107a degranulation marker expression and facilitated cytotoxicity of target tumor cells by both Vδ1 and Vδ2 T cells. The agonistic activity of TAVO307 × VHH371 depended on the ligation of both the γδ T cells and tumor cells, since control antibodies incapable of bridging the ligation of these cells did not result in γδ T cell agonism. Besides, the agonism of TAVO307 × VHH371 demonstrated potent picomolar EC_50_ yet displayed a paradoxical loss of activity at very high drug concentrations (hook effect), a phenomenon reported for other TCEs, underscoring the need to select dosing regimens that maintain exposure within an optimal therapeutic window. The TAVO307 × VHH371 TCE recruitment of both Vδ1 and Vδ2 T cells in tumor cell cytotoxicity could be advantageous over just recruiting a single γδ T cell subset. Recruiting Vδ1 T cells, which are present in large quantity in the intestinal mucosa, could result in more rapid and efficacious tumor elimination in GI tract tumors. In addition, recruiting the Vγ9Vδ2 cytotoxic T cells dominantly present in blood circulation could help to eliminate tumor cells in CDH17-associated metastasis. However, Vδ1 T cells are heterogeneous cell populations, due to Vδ1 chain pairing with different Vγ chains, functioning as either anti-tumor, pro-tumor, or tumor microenvironment regulation [59–61]. Although our TAVO307 × VHH371 TCE mediated the killing of CDH17-expressing tumor cells by expanded Vδ1 T cells, further studies would be needed to evaluate the effects of TCE on different subtypes of Vδ1 T cells.

However, a persistent challenge in γδ T cell–based therapies is the limited endogenous repertoire, phenotypic exhaustion, or suppression of γδ T cells in cancer patients. Nonetheless, targeted human γδ T cells-driven tumor cytotoxicity and inflammatory cytokine release could result in such cells acquiring professional APC functions by processing and cross-presenting tumor antigens to CD4^+^ and CD8^+^ T cells, which could then shape downstream αβ T cell priming [62]. Therefore, although limited in quantity, the γδ T cells could act as “innate-adaptors” to coordinate fast, innate-style tumor control with the generation of adaptive sustained anti-tumor immunity.

To overcome the potential poor persistence of the limited number of γδ T cells in cancer treatment, we engineered a fusion of an attenuated IL-15 with the sushi domain of IL-15 receptor α onto the TAVO307 × VHH371 TCE. This IL-15 domain was an attractive immunotherapy design by enhancing the proliferation, cytotoxicity, and Th1 polarization of γδ T cells [63]. Our data demonstrated that the incorporation of IL-15 fusion on TAVO307 × VHH371 TCE stimulated the proliferation and activation of γδ T cells in a γδ TCR-binding-dependent manner. With CDH17-mediated tumor targeting, IL-15 could augment local γδ T cell expansion and persistence in tumor cytotoxicity. However, careful titration of IL-15 activity was required to avoid systemic cytokine toxicity or off-target expansion of other immune subsets. The engineering of a mutant IL-15 domain, with a 10–100-fold weaker activation relative to native IL-15, resulted in a molecule that did not stimulate the proliferation and activation of αβ T cells or NK cells at the same concentrations that stimulated γδ T cells. On the other hand, a TCE with IL-15 fusion could activate the γδ T cells independent of ligation to tumor cells, which could lead to off-target cytotoxicity to neighboring cells (i.e. NK ligands that can be recognized by γδ TCR). Therefore, further fine-tuning of the IL-15 activity or conditional tumor microenvironment activation would be needed to boost γδ T cell quantity and persistence for better tumor cytotoxicity while avoiding toxicity associated with off-target activation of different types of T cells and NK cells. This needs to be evaluated in cell-based assays with multiple PBMC donors as well as in animal studies.

In summary, our study positioned CDH17 to be a versatile and clinically tractable target in GI cancers. By linking the precision of ADC technology with the adaptability of γδ T cell engagement, we provided a foundation for complementary dual-faceted therapeutic strategies. ADCs could deliver potent cytotoxicity independent of the immune context, while γδ TCEs could recruit immune effector mechanisms to generate durable responses and immunologic memory. As a future direction, it will be intriguing to study whether combining ADCs with γδ TCEs (either sequentially or concurrently) could result in synergistic tumor killing, epitope spread, and resistance mitigation. Indeed, combining ADCs with immunotherapy, mostly PD-1/PD-L1 immune checkpoint inhibitors, has become a paradigm for cancer therapies [64]. ADC-induced immunogenic cell death could release antigens to further stimulate T cell responses. With further optimization and translational rigor, these modalities could improve outcomes for patients with GI malignancies.

## Supplementary Material

ABT-2025-047_supplemental_figures_revised_tbag012

## Data Availability

The data underlying this article are available in the article and in its online supplementary material. Further inquiries can be directed to the corresponding author.

## Abbreviations

ADC, antibody drug conjugate; CAR, chimeric antigen receptor; CDH17, cadherin-17; CRS, cytokine release syndrome; DAR, drug to antibody ratio; DPBS, Dulbecco’s phosphate-buffered saline; GI, gastrointestinal; HC, heavy chain; HIC, hydrophobic interaction chromatography; ICANS, immune effector cell-associated neurotoxicity syndrome; IEL, intraepithelial lymphocyte; IFN-γ, interferon γ; IL-15, Interleukin-15; LC, light chain; MFI, mean fluorescence intensity; MHC, major histocompatibility complex; MMAE, monomethyl auristatin E; MMAF, monomethyl auristatin F; NK, natural killer; PBMC, peripheral blood mononuclear cell; SEAP, secreted embryonic alkaline phosphatase; SEC, size exclusion chromatography; SD, sushi domain; TCE, T cell engager; TCEP, Tris(2-carboxyethyl)phosphine; TCR, T cell receptor; TMB, 3,3′,5,5′-tetramethylbenzidine.

## Animal research statement

All procedures involving animal care, handling, and treatment complied with the guidelines of the Institutional Animal Care and Use Committee (IACUC) of Suzhou Charles River Accelerator and Development Lab (Approved Protocol: P202302160002-20240901).
