# Supplementary material for: Novel ADC and γδ T cell engager targeting CDH17 for the therapy of gastrointestinal cancers
Source: Antib Ther. 2026 Mar 12;9(3):230–47. doi: 10.1093/abt/tbag012 (PMC13317742; doi:10.1093/abt/tbag012)
Supplement: ABT-2025-047_supplemental_figures_revised_tbag012 [file abt-2025-047_supplemental_figures_revised_tbag012.docx]

Supplemental Figure 1


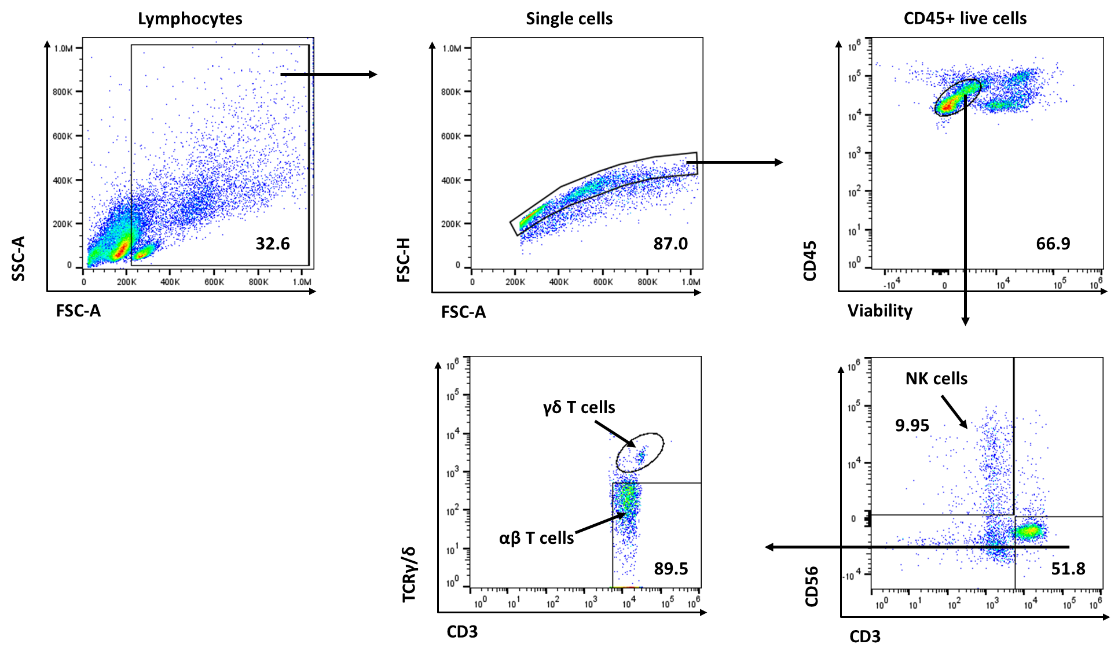


Supplemental Figure 1. Representative two-parameter density flow cytometry plots of PBMCs showing the gating strategy to define αβ T cells, γδ T cells and NK cells. The lymphocyte population of PBMC were identified and gated by forward scatter (FSC) and side scatter (SSC). From lymphocyte, single cells were gated and then CD45^+^ live cells were further identified, of which NK cells, αβ T cells and γδ T cells were gated by the expression of CD56, CD3 and TCRγδ markers.

Supplemental Figure 2

1. **(B)**


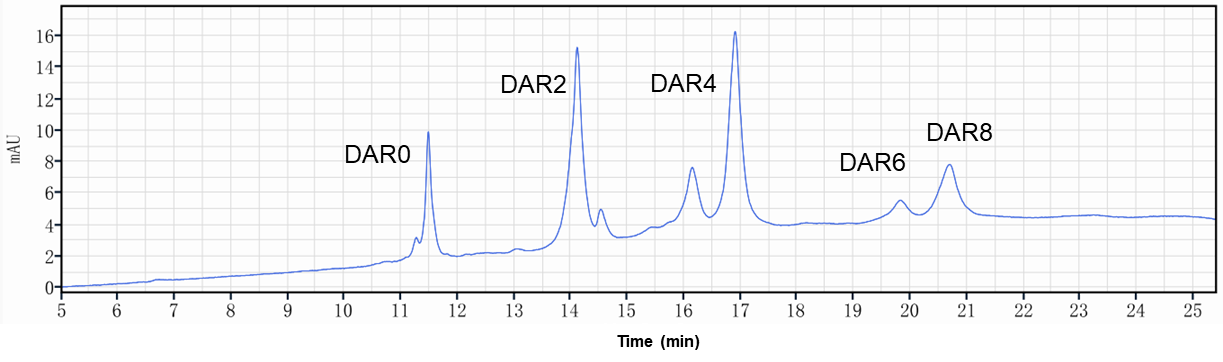

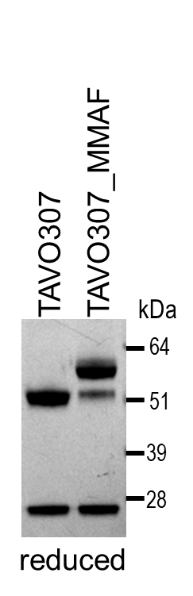


**(C)**


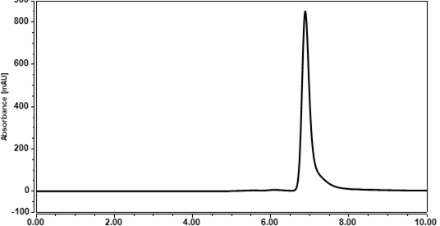


Supplemental Figure 2. Characterization of CDH17 ADC. (A). Gel images of protein bands of TAVO307 and TAVO307_MMAF subjected to SDS-PAGE analysis under reduced condition. (B) HIC analysis of TAVO307_MMAE. The Y axes units are in absorbance values at 280 nm. The x axes units are retention time in minutes. (C). SEC analysis of TAVO307_MMAE. The Y axes units are in absorbance values at 280 nm. The x axes units are retention time in minutes.

Supplemental Figure 3

**(A)** **(B)**


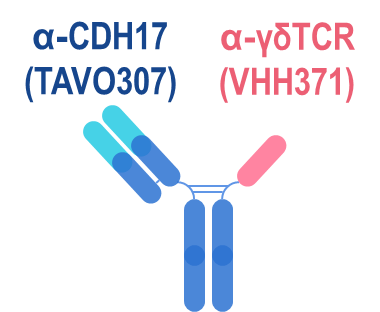

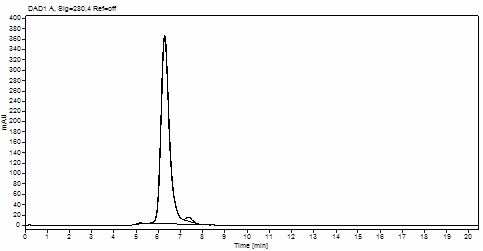


**(C)** **(D)**


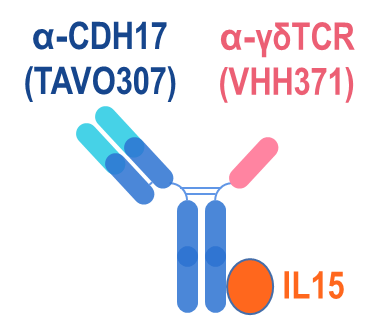

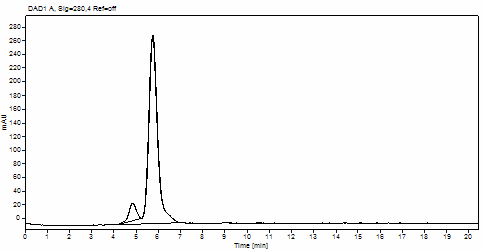


**(E)**

Supplemental Figure 3. Characterization of CDH17 γδTCE. (A). Schematic drawing of TAVO307 x VHH371. (B). SEC analysis of TAVO307 x VHH371. The Y axes units are in absorbance values at 280 nm. The x axes units are retention time in minutes. (C). Schematic drawing of TAVO307 x VHH371_IL15. (D). SEC analysis of TAVO307 x VHH371_IL15. The Y axes units are in absorbance values at 280 nm. The x axes units are retention time in minutes. (E). Flow cytometry based binding assays showed antibodies binding to AsPC-1 cells. MFI were plotted against the concentrations of the antibodies. Experiments were performed in duplicate with data reported as mean ± SD.

Supplemental Figure 4

**(A)** **(B)**

Supplemental Figure 4. IL-15 functional assays. (A). Reporter gene activation driven by TAVO307 x VHH371 and TAVO307 x VHH371_IL15 antibodies in HEK-Blue CD122/CD132 reporter assays. The folds of reporter gene activation over the case without antibody added were plotted against the concentrations of testing antibodies. Experiments were performed in duplicate with data reported as mean ± SD. (B). CTLL-2 cell proliferation driven by TAVO307 x VHH371 and TAVO307 x VHH371_IL15 antibodies in CTLL-2 cell proliferation assays. The folds of cell proliferation over the case without antibody added were plotted against the concentrations of testing antibodies. Experiments were performed in duplicate with data reported as mean ± SD.

Supplemental Table 1

|  | **native**  EC_50_ (pM) | **S7T / I68K**  EC_50_ (pM) | fold of potency attenuation |
| --- | --- | --- | --- |
| HEK-Blue IL-15 reporter assay | 1.3 | 12.8 | 10 |
| CTLL-2 cell proliferation assay | 2.5 | 235.3 | 94 |
| NK cell activation assay | 40.9 | 1701.6 | 42 |
| CD8 T cell activation assay | 85.0 | 4527.7 | 53 |

Supplemental Table 1. The native IL-15 or IL-15 with the S7T and I68K mutations along with the sushi domain (SD) of IL-15 receptor α were engineered at the heavy chain C-termini of a null antibody as fusion proteins. The IL-15-mediated functional activities were assessed in the HEK-Blue IL-15 reporter assay, CTLL-2 cell proliferation assay, NK cell activation assay and CD8 T cell activation assay. The potencies of IL-15 activities in terms of EC_50_ were calculated and the fold of potency attenuation due to S7T/I68K mutations were presented.
